# Normal cerebral oxygen consumption and lactate levels in patients with Alzheimer’s disease and Lewy body dementia

**DOI:** 10.1007/s11357-025-01658-x

**Published:** 2025-04-24

**Authors:** Christian Sandøe Musaeus, Gunhild Waldemar, Troels Wesenberg Kjær, Birgitte Bo Andersen, Peter Høgh, Steen Gregers Hasselbalch, Ulrich Lindberg, Kristian Steen Frederiksen, Henrik Bo Wiberg Larsson, Mark B. Vestergaard

**Affiliations:** 1 Danish Dementia Research Centre (DDRC), Department of Neurology, Copenhagen University Hospital –Rigshospitalet, Inge Lehmanns Vej 8, 2100 Copenhagen, Denmark; 2 Department of Clinical Medicine, University of Copenhagen, Blegdamsvej 3B, 2200 Copenhagen, Denmark; 3 Aalborg University, Fredrik Bajers Vej 7, Aalborg East, Denmark; 4 Regional Dementia Research Centre, Department of Neurology, Zealand University Hospital, Vestermarksvej 11, 4000 Roskilde, Denmark; 5 Functional Imaging Unit, Department of Clinical Physiology and Nuclear Medicine, Copenhagen University Hospital - Rigshospitalet, Valdemar Hansens Vej 13, 2600 Glostrup, Denmark

**Keywords:** Hypometabolism, Metabolism, Lactate, Oxygen, Perfusion, Alzheimer’s disease

## Abstract

Brain metabolism is reduced in patients with dementia disorders, as demonstrated by hypometabolism on 2-deoxy-2-[^18^F]fluoroglucose ([^18^F]FDG) positron emissions tomography. A contributing factor to the hypometabolism could be decreased cerebral blood flow (CBF) leading to a state of subtle hypoperfusion‐induced tissue hypoxia causing a reduced brain oxygen metabolism and consequently elevated brain lactate. In the current exploratory study, we investigated brain lactate, global and regional CBF, and global cerebral metabolic rate of oxygen (CMRO_2_) in patients with Alzheimer’s disease (AD) and dementia with Lewy bodies (DLBs). We hypothesized that the patients demonstrate a state of tissue hypoxia with reduced CMRO_2_ and elevated brain lactate concentration. Participants included 24 AD patients, 10 DLB patients, and 15 healthy controls. MR spectroscopy measured lactate in the precuneus and occipital lobe. Global CBF and venous oxygen saturation (for CMRO2 calculation) were assessed using phase-contrast and susceptibility-based oximetry MRI, respectively. Regional CBF was measured with ASL-MRI. We observed no significant difference in either brain lactate or CMRO_2_ between groups. The regional CBF in precuneus was significantly lower in AD compared to HC; however, this hypoperfusion was not associated with a higher lactate concentration. The lack of a difference in CMRO_2_ or lactate concentration between patients and controls suggests that the hypometabolism observed in patients with AD and DLB may reflect structural neurodegeneration and not a state of tissue hypoxia. The local decrease of CBF in precuneus in patients with AD may be due to a lower CBF demand due to neurodegeneration.

## Introduction

Changes in brain glucose metabolism can be detected by the early disease phase in patients with neurodegenerative diseases [1, 2]. Most commonly, this has been assessed using 2-[^18^F]fluoro- 2-deoxy-d-glucose ([^18^F]FDG) positron emission tomography (FDG-PET) showing hypometabolism. Hypometabolism has been found in patients with dementia of different etiologies including Alzheimer’s disease (AD) [3] and Lewy body dementia (DLB) [4]. It is possible that the reduced glucose consumption in dementia may be associated with a state of brain tissue hypoxia, causing hypometabolism.

One possible explanation for brain tissue hypoxia in AD could be cerebral vascular dysfunction. Reduced regional cerebral blood flow (rCBF) and capillary blood flow disturbances, potentially caused by arteriosclerosis or arterial stiffening, may hinder sufficient blood and oxygen delivery [5, 6]. Reduced rCBF has been found in both patients with AD and DLB, particularly in brain regions commonly affected by these conditions, such as the hippocampus and insula [7, 8]. A recent theory suggests that tissue hypoxia arises not only from reduced blood flow but also from uneven distribution of blood flow and transit times within the capillary [9]. These variations can impair oxygen diffusion, causing regional hypoxia despite adequate overall cerebral blood flow. Brain hypoxia may contribute to hypometabolism, reduced beta-amyloid clearance, inflammation, and oxidative stress due to reactive oxygen species (ROS) [10–12].

If tissue hypoxia is present, the cerebral metabolic rate of oxygen (CMRO_2_) would likely decrease, while brain lactate levels would rise. Previous studies on CMRO_2_ and brain lactate in AD or DLB have examined these factors separately, showing mixed results. CMRO_2_ studies show decreased or unchanged levels in AD compared to controls [13–17], with discrepancies potentially due to methodological differences, such as how atrophy is accounted for. Measuring lactate using magnetic resonance spectroscopy (MRS) studies found elevated lactate in patients with AD [18, 19]. Cerebrospinal fluid (CSF) lactate studies have resulted in mixed results, reporting either decreased levels [20] or no difference [21] in AD versus controls. However, CSF lactate may not fully reflect brain tissue concentrations, as lactate can be removed through blood, CSF, or local metabolism [22].

The aim of the present exploratory study was to examine the state of brain tissue hypoxia in patients with AD or DLB by measuring both CBF, CMRO_2_, and brain lactate concentration using MRI and MR spectroscopy techniques. Accompanying CMRO_2_ measurement with simultaneous brain lactate assessment provides a description of potential oxygen metabolism deficits. Brain lactate production is highly reactive to hypoxic exposure [23] and will in combination with low CMRO_2_ and CBF provide an indication for tissue hypoxia. Additionally, we measured *N*-acetyl aspartate (NAA), a biomarker for neuronal density and function [24], by MRS, which has previously been shown to be decreased in patients with AD. This will provide information of neuronal loss in the patients. The study was performed in the precuneus, as this is a key node of the default mode network [25] and shows early neuropathological abnormalities [26] with decreased glucose metabolism [27]. To understand if any changes in metabolism is specific to the precuneus, we also investigated whether increased lactate level could be identified in the occipital lobe as occipital hypometabolism is present even in the prodromal phase in patients with DLB [28] but not in amnestic AD. Lastly, we investigated whether an association could be found between white matter hyperintensities (WMHs) and global rCBF to understand if it was associated with vascular load.

We hypothesized an elevated brain lactate and reduced CMRO_2_, which would be associated with reduced CBF in both AD and DLB patients. We further hypothesized that an elevated lactate concentration would be more pronounced in the precuneus for patients with AD and more pronounced in the occipital lobe for patients with DLB due to predominant topographic involvement of pathology in these two types of dementia.

## Methods

### Participants

In the current study, we included patients with AD and DLB as well as healthy controls (HCs). All patients with either AD or DLB had mild to moderate dementia. Participants were recruited from the Memory Clinic at Copenhagen University Hospital—Rigshospitalet, Copenhagen, and the Memory Clinic at Zealand University Hospital, Roskilde. The patients with AD met the NIA-AA criteria for probable AD with amnestic presentation [29] and the patients with DLB met the Consortium criteria for DLB [30] both determined based on a consensus conference. This consensus conference was conducted after clinical evaluation and evaluated the clinical findings including structural imaging and, in most instances, 18 F-FDG-PET and neuropsychological evaluation. A full description of the diagnostic evaluation can be found in previous studies [31, 32]. The inclusion and exclusion criteria for patients with AD [31, 33] and DLB [32] as well as the HC [31, 32] can be found in the previous studies. The data presented in this manuscript is from a larger study on the patients primarily focusing on electroencephalography (EEG) measurements, which has been previously published [31, 32]. Data on brain anatomy and hippocampal perfusion from the MRI scans has been used in the previously published studies as supporting data for the EEG investigation [31–34]. The data on brain oxygen and lactate metabolism have not been presented before.

The study was approved by the Capital Region Ethics Committee (H- 17035751), and by the Danish Medicines Agency (2,017,112,288), and registered at the Data Protection Agency (P- 2021–866). All participants gave written and oral informed consent before participating in the study. The study is registered at clinicaltrials.gov (NCT04436341).

### Study design

In this cross-sectional study, all patients had an initial visit to the Memory Clinic at Copenhagen University Hospital where informed consent was obtained followed by assessment of medical history, a physical and neurological examination, and the Mini-Mental State Examination (MMSE) (for assessment of global cognitive function). After the initial examination, the patient underwent an MRI scan and completed the Functional Assessment Questionnaire IADL (FAQ IADL) [35] (to assess everyday function), and the neuropsychiatric inventory (NPI) [36] (to assess behavioral and psychological symptoms).

### Magnetic resonance imaging

**Fig. 1:**
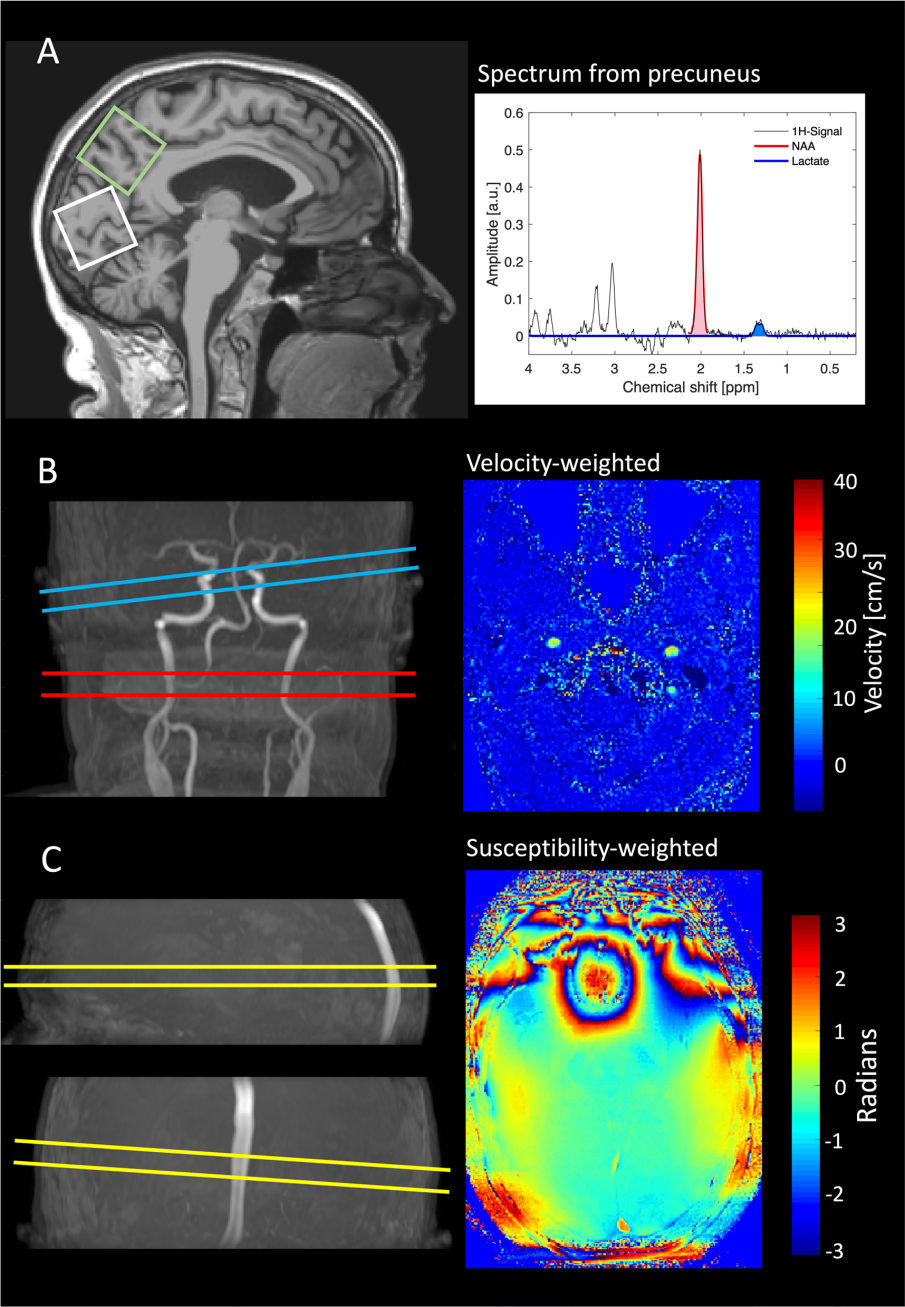
MR methods. **A** MR spectroscopy was used to measure cerebral lactate and *N*-acetylaspartate (NAA) concentrations in precuneus (green square) and the occipital lobe (white square). **B** Phase-contrast MRI measured global cerebral blood flow by obtained velocity-weighted images perpendicular to the carotid arteries (red plane) and the basilary artery (blue plane), and **C** susceptibility-based oximetry MRI technique was used to measure cerebral venous oxygen saturation of the blood leaving the brain through the sagittal sinus enabling calculation of cerebral metabolic rate of oxygen

The MRI scans were conducted on a 3 T Philips Achieva dStream scanner (Philips Medical Systems, Best, The Netherlands, software release 5.4.1), using a 32-channel phased array receive head coil. In-depth description of the MRI sequences and investigation of the reproducibility of the calculated parameters have been provided previously [37, 38]. During the MRI scans, the heart rate and peripheral oxygen saturation were monitored using a Veris Monitor system (Medrad, Pittsburgh, Pennsylvania [39]. See Fig. 1 for an overview of the methods used in the study including magnetic resonance spectroscopy, phase contrast mapping, and CMRO_2_ based on susceptibility-based oximetry.

### Structural images

Structural images were acquired with a sagittal three-dimensional T1-weighted magnetization prepared rapid acquisition gradient echo (MPRAGE) sequence. The acquisition parameters were: matrix size 256 × 255, 155 slices, voxel size 1.1 × 1.1 × 1.1 mm^3^, field of view (FoV) 263 × 280 × 171 mm^3^, TR 6.9 ms, TE 2.82 ms, and flip angle 9°. The brains were segmented with the FreeSurfer software package [40] using the recommended procedure (Martinos Center for Biomedical Imaging, Massachusetts, USA).

White matter lesion load was assessed by T2-weighted brain images measured using a fluid attenuated inversion recovery (FLAIR) sequence with the following acquisition parameters: matrix size 384 × 384, 35 slices, voxel size 0.6 × 0.6 × 3.5 mm^3^, FoV 230 × 134 × 182 mm^3^, repetition time (TR) 11,000 ms, echo time (TE) 125 ms, and flip angle 90°. Evaluation of white matter lesion load was performed using Fazekas score [39] with visual rating of the MRI flair sequence. The rating was performed by CSM and HBWL.

### Global cerebral blood flow

The global cerebral blood flow (CBF) was obtained using velocity sensitive phase contrast mapping (PCM) MRI [41, 42]. Blood velocity contrast maps were acquired by a turbo field echo sequence. The acquisition parameters were: matrix size 320 × 320, voxel size 0.75 × 0.75 mm^2^, one slice, slice thickness 8 mm, FoV 240 × 240 mm^2^, TR 12 ms, TE 7.4 ms, flip angle 10°, velocity encoding (VENC) = 100 cm/s, 5 repeated measurements. Two measurements were acquired, one from an imaging plane perpendicular to the carotid arteries and another from an imaging plane perpendicular to the basilar artery. The blood flow in both internal carotids and the basilar artery was calculated by multiplying the mean blood velocity by the cross-sectional area from regions of interest defining each vessel. Delineation of the vessels was performed using custom-built and publicly available software (see data availability). The global CBF was calculated as the sum of blood flow in the internal carotid arteries and the basilar artery. The global CBF normalized to brain weight was calculated by dividing the total blood flow from each artery to the total brain weight (per 100 g), thus obtaining CBF values in ml/100 g/min. Brain weight was estimated from the segmentation of the structural MRI images and assuming a brain density of 1.05 g/ml. The values presented in the current study are slightly lower than in a previous study [34] due to a minor calculation error in former studies, which did not affect the conclusion.

### Regional cerebral blood flow

A pseudo continuous ASL (PCASL) sequence with Look-Locker Echo Planar Imaging was chosen. The labeling plane was placed across the neck 9 cm beneath the center of the imaging slab, and the labeling duration was 1650 ms. The acquisition parameters were as follows: matrix size 64 × 64, voxel size 3.44 × 3.44 mm^2^, 13 slices, slice thickness 6.6 mm, FoV 220 × 220 × 85 mm^3^, TE 10.8 ms, TR was 300 ms, Look-Locker flip angle 40°, slice acquisition duration 22 ms, SENSE factor 2.3. The post-labeling delays were set at [100, 400, 700, 1000, 1300, 1600, 1900 ms]. Each ASL pair (label and control) took 8 s. After each ASL scan, a single-equilibrium magnetization scan (M0) was acquired with the same parameters as the previously described ASL images except for a TR of 10,000 ms.

ASL images were quantified using BASIL in FSL (FMRIB software library, version 6.0.5.1, www.fmrib.ox.ac.uk) with quantification [43] and fitting of the macrovascular compartment [44]. The initial prior of bolus arrival time was adjusted to account for the delayed arrival as seen in our sample, which resulted in a selected arrival time prior 1.6 s. Lastly, the ASL data were registered to the structural scan. The ASL quantification was not corrected for differences in hematocrit values.

To quantify rCBF in the precuneus, we first extracted all the values within the precuneus ROI segmented with FreeSurfer and then removed the voxels with zero values as these were assumed to be contaminated by CSF. Afterwards, any values more than two standard deviations above the mean were removed since they were assumed most likely to represent arteries. Finally, the value was normalized to the global mean CBF as measured with PCM by dividing the regional CBF in precuneus with the global mean CBF.

### Cerebral metabolic rate of oxygen

The global mean cerebral metabolic rate of oxygen was calculated using the Fick principle:

1 $${\mathrm{CMRO}}_{2}=\mathrm{Hgb}\bullet \mathrm{CBF}\bullet \left({\mathrm{SaO}}_{2}-{\mathrm{SvO}}_{2}\right)$$

CBF was measured by PCM MRI as described above. Hgb was assumed based on a previous study on hemoglobin concentration in aging subjects [45]. SaO_2_ was measured by pulse oximetry. The oxygen saturation of the venous blood (SvO_2_) in sagittal sinus leaving the brain was measured using the susceptibility-based oximetry (SBO) MRI technique [46]. A dual-echo gradient-echo sequence was used to acquire susceptibility-weighted brain images (matrix size 320 × 320, voxel size 0.69 × 0.69 mm^2^, 1 slice, slice thickness 8 mm, FoV 220 × 190 mm^2^, TR 23.1 ms, TE1/TE2 8.16/17.83 ms, flip angle 30°, SENSE factor 2, 5 repeated measurements). The imaging plane was placed orthogonal to the sagittal sinus to minimize partial volume contamination. Post-processing and calculation of SvO_2_ values were performed by in-house developed and publicly available software (see data availability).

### Lactate and N-acetyl aspartate

The concentrations of lactate and NAA were measured in precuneus and the occipital lobe with magnetic resonance spectroscopy (MRS). A single-voxel water-suppressed point-resolved 1H-spectroscopy (PRESS) sequence was used (voxel size 30 × 35 × 30 mm^3^, TR 2000 ms, TE 288 ms, 176 averages, 1024 complex data points). A water un-suppressed spectrum was additionally acquired to quantify metabolite concentration using the water peak as reference. The water concentration in the MRS-voxel was calculated based on the tissue composition (grey matter, white matter, and CSF) based on segmentation of the high-resolution structural images [47]. Post processing and quantification of concentration were performed using LCModel (LCModel Version 6.3 - 1 F, Toronto, Canada). The concentrations were corrected for T_2_ decay based on literature values (T_2_^H2O^ 95 ms, T_2_^NAA^ 247 ms, T_2_^Lac^ 240 ms) [48, 49].

### Statistics

The statistical analyses were performed in RStudio (v1.2.1335). When comparing age, education, and MMSE test scores, we performed an ANOVA across the three groups (AD, DLB, and HC). Chi-squared test was used to compare sex between the groups. To test for differences in lactate and NAA concentration between the patient and HC, an ANCOVA model was used. The ratio of grey and white matter in the MRS was added as nuisance variable to the model to correct for different tissue compositions and brain atrophy between participants. ANOVA models were used to test for differences in CBF, and CMRO_2_ between patients and controls. rCBF values were log-transformed due to non-normal distribution of the residuals. If the ANOVA or ANCOVA was significant, a post hoc comparison was conducted using the Tukey test with single‐step adjustment as implemented in the *multcomp* toolbox.

To understand if there was a difference in global CBF between Fazekas groups (0–3), we divided the participants into groups depending on their Fazekas score.

To test if elevated lactate in precuneus could be a result of hypoperfusion, we used a linear regression model between normalized rCBF and lactate concentration in precuneus.

## Results

### Participant characteristics

A total of 25 patients with AD, 10 patients with DLB, and 15 HCs were included in the study. See Table 1 for participant characteristics.

**Table 1:** Participant characteristics

	Healthy controls	Alzheimer’s disease	Lewy body dementia	*p* value
Number of participants	15	25	10	
Age, mean (SD)	69.5 (7.93)	70.3 (7.79)	69.9 (8.49)	0.959
Males/females	8/7	15/10	10/0	0.036
Education in years, mean (SD)	15.2 (2.04)	14.2 (3.08)	14.3 (2.91)	0.534
Cholinesterase inhibitor, *n* (%)	0	24 (96%)	9 (90%)	
Levodopa, *n* (%)	0	0	3 (30%)	
SSRI/SNRI, *n* (%)	1 (7%)	4 (16%)	2 (20%)	
MMSE, mean (SD)	29.3 (0.88)	23.4 (3.29)	26.7 (1.83)	< 0.001
NPI		4.83 (4.06)	7.67 (7.19)	
ADL		14.28 (5.78)	12.67 (5.79)	
Amyloid positive, *n*/total^a^		18/20	4/5	
Phosphorylated tau (pg/ml), mean (SD)^b^		82.41 (31.19)	39.2 (10.08)	
Total tau (pg/ml), mean (SD)^b^		568.88 (242.34)	222.8 (92.07)	

*SSRI/SNRI* selective serotonin reuptake inhibitor/serotonin and noradrenaline reuptake inhibitor, *MMSE* Mini-Mental State Examination, *ADL* Activities of Daily Living, *NPI* Neuropsychiatric Inventory, *SD* Standard deviation

^a^Amyloid positive was defined by either a value below the year-corrected cut-off using CSF amyloid or positive amyloid PIB-PET at the time of diagnosis in patients who had undergone testing in either one of these modalities (AD *n* = 20, DLB *n* = 5)

^b^Both phosphorylated tau (cut-off < 60) and total tau (cut-off < 400) were measured as part of the initial diagnosis

### Lactate and N-acetyl aspartate

**Fig. 2:**
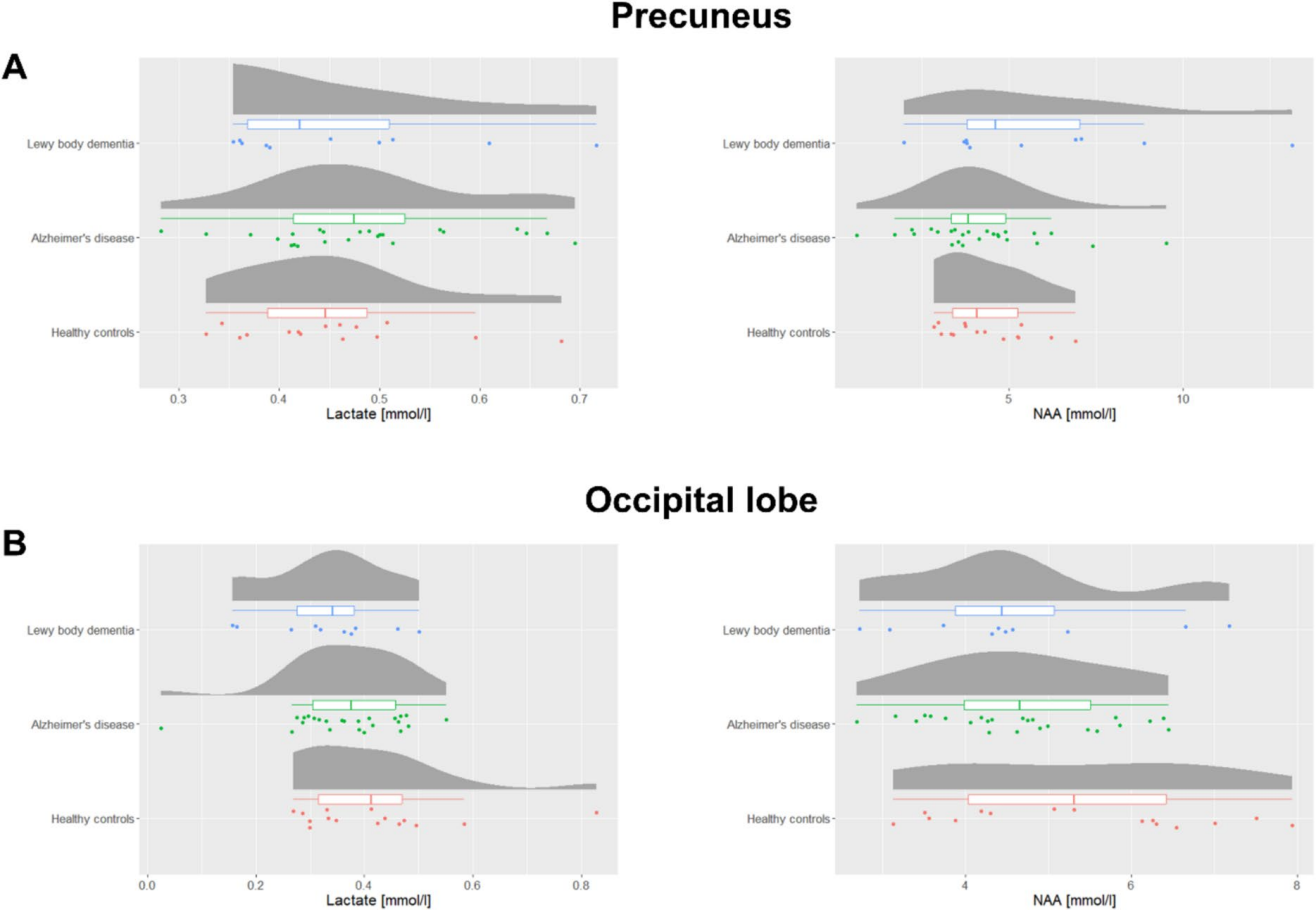
Raincloud plots showing the lactate concentrations (on the left) and *N*-acetylaspartate (NAA) concentrations (on the right) for Lewy body dementia (*n* = 10), Alzheimer’s disease (*n* = 25), and healthy controls (*n* = 15) in the **A** the precuneus and **B** the occipital lobe. The boxplots indicate the median, interquartile range while the “whiskers” extend to the largest and smallest data points within 1.5 times the interquartile range from the third quartile and first quartile, respectively. A significant difference in *N*-acetylaspartate concentration was found between Alzheimer’s disease and Lewy body dementia (*p* = 0.034)

In the precuneus, we did not find a significant difference in lactate between AD, DLB, and HC (*p* value: 0.69, *F*-value: 0.38). For NAA, a significant difference was seen between the groups (*p* value: 0.044, *F*-value: 3.35). In the post hoc analysis, we found a significant lower NAA concentration in AD (mean (SD): 4.2 mmol/l (1.9)) compared to DLB (mean (SD): 5.9 mmol/l (3.3)) with a *p* value of 0.034 (Fig. 2).

In the occipital lobe, no significant difference in either lactate (*p* value: 0.079, *F*-value: 2.70) or NAA (*p* value: 0.24, *F*-value: 1.47) was found between the groups (Supplementary Fig. 1).

### Global cerebral metabolic rate of oxygen

**Fig. 3:**
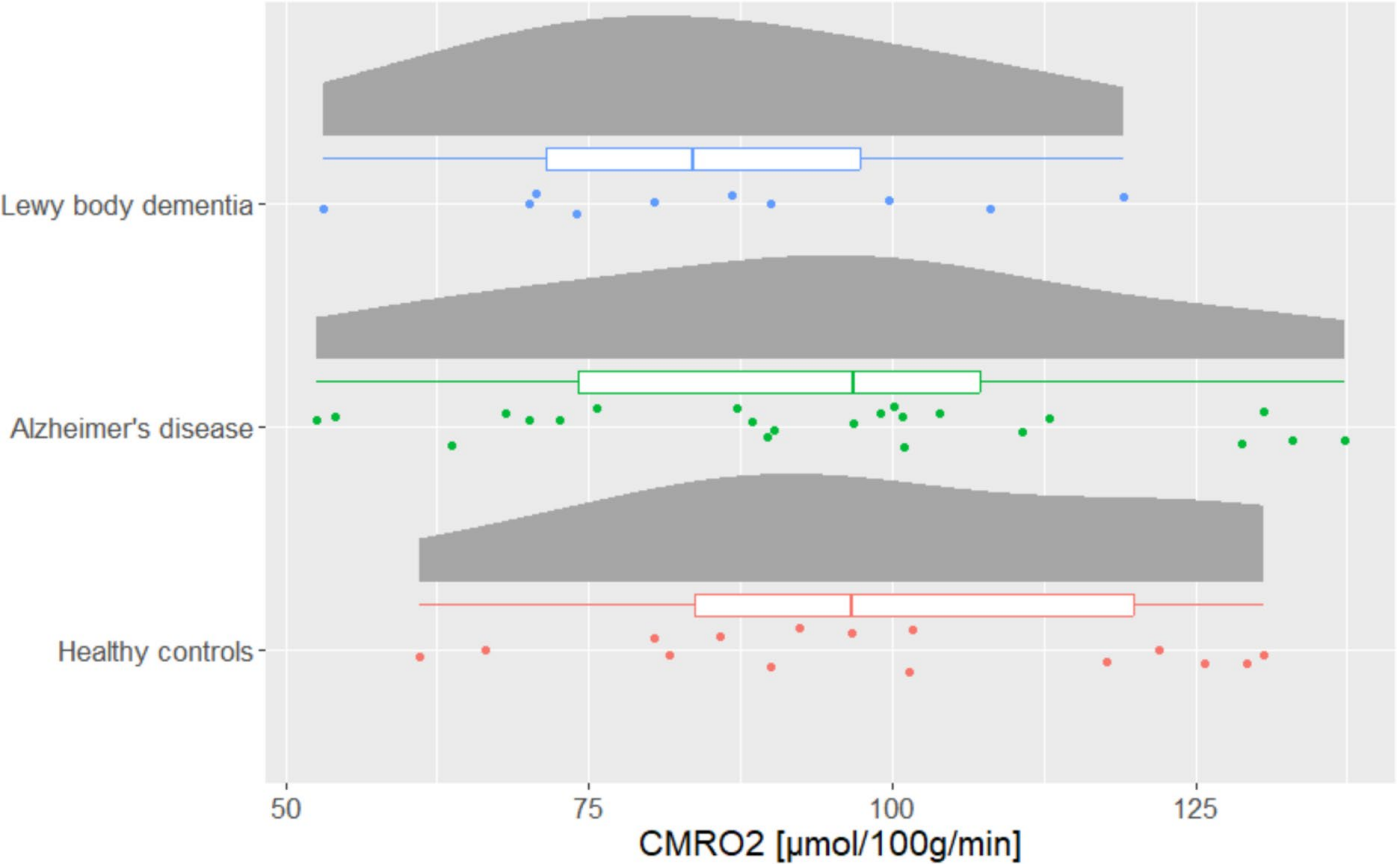
Raincloud plots showing the cerebral metabolic rate of oxygen (CMRO_2_) for patients with Lewy body dementia (*n* = 10), Alzheimer’s disease (*n* = 25), and healthy controls (*n* = 15). The boxplots indicate the median, interquartile range while the whiskers extend to the largest and smallest data points within 1.5 times the interquartile range from the third quartile and first quartile, respectively. No significant differences were found between the groups (*p* = 0.35)

No significant difference in CMRO_2norm_ (*p*-value = 0.35, *F*-value = 1.07) was found between AD (mean (SD): 94.2 mmol/100 g/min (23.5)), DLB (mean (SD): 85.2 mmol/100 g/min (19.8)), and HC (mean (SD): 98.9 mmol/100 g/min (22.3)) (see Fig. 3).

### Global cerebral blood flow

**Fig. 4:**
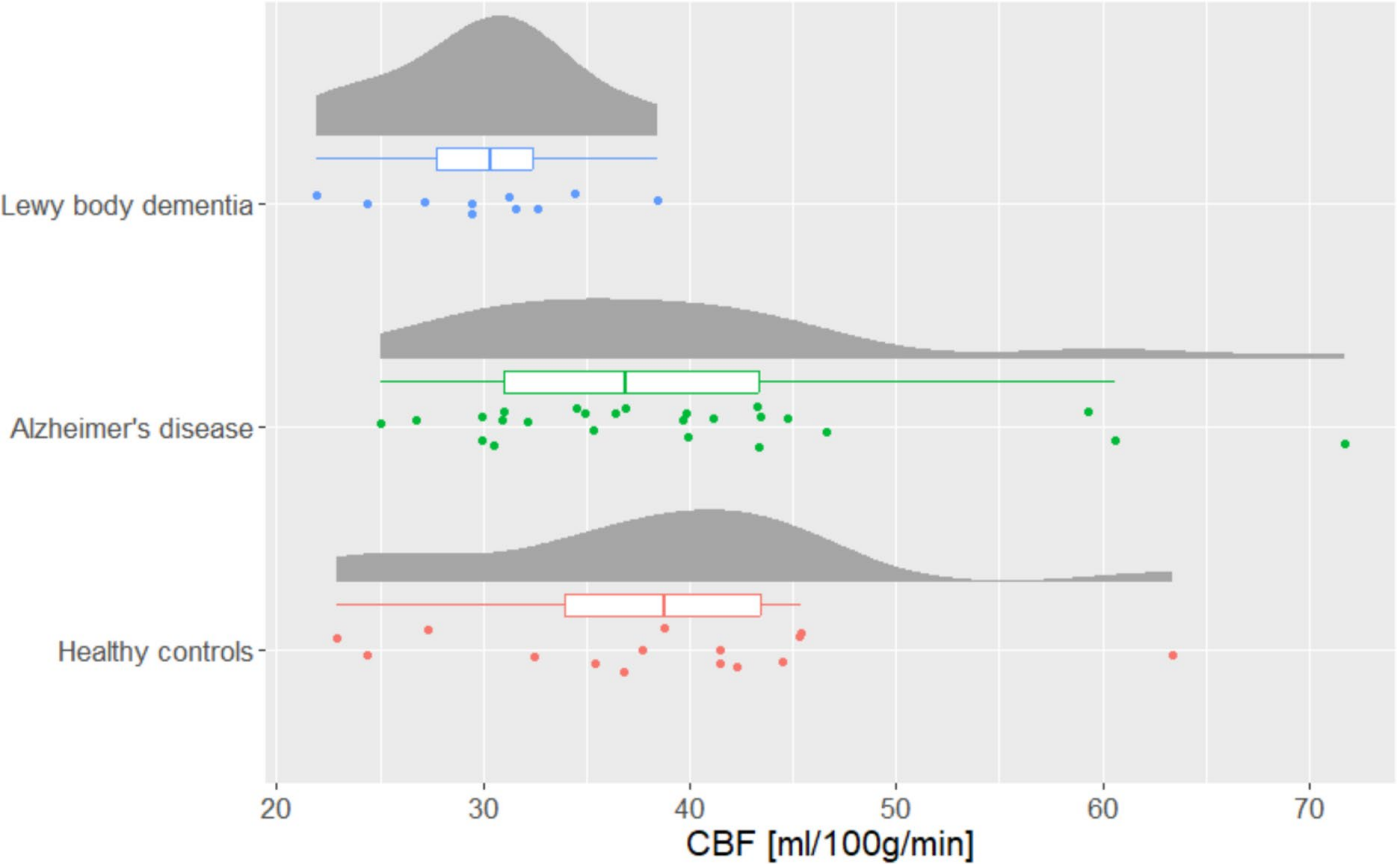
Raincloud plot showing the global cerebral blood flow (CBF) for patients with Lewy body dementia (*n* = 10), Alzheimer’s disease (*n* = 25), and healthy controls (*n* = 15). The boxplots indicate the median, interquartile range while the whiskers extend to the largest and smallest data points within 1.5 times the interquartile range from the third quartile and first quartile, respectively. A significantly lower global CBF was seen in Lewy body dementia compared to Alzheimer’s disease (*p* = 0.020) but only borderline between healthy controls and Lewy body dementia (*p* = 0.060)

Global CBF was significantly (*p* value = 0.023, *F*-value = 4.10) different between AD (mean (SD): 39.5 ml/100 g/min (11.0)), DLB (mean (SD): 30.1 (4.79)) and HC (mean (SD): 38.6 ml/100 g/min (10.0)) (Fig. 4). The post hoc analysis found a significantly lower global CBF in DLB compared to AD (*p* value: 0.020) but only borderline between HC and DLB (*p* value: 0.060) but not between AD and HC.

We did not find a significant difference in CBF depending on the Fazekas score for deep white matter hyperintensities (*p* value: 0.20) although lower global CBF was observed in the patients with higher Fazekas score (F0, mean (*n*): 38.6 (1), F1, mean (*n*): 38.5 (31), F2, mean (*n*): 34.8 (14), F3, mean (*n*): 32.62 (4)).

### Regional CBF and lactate in precuneus

**Fig. 5:**
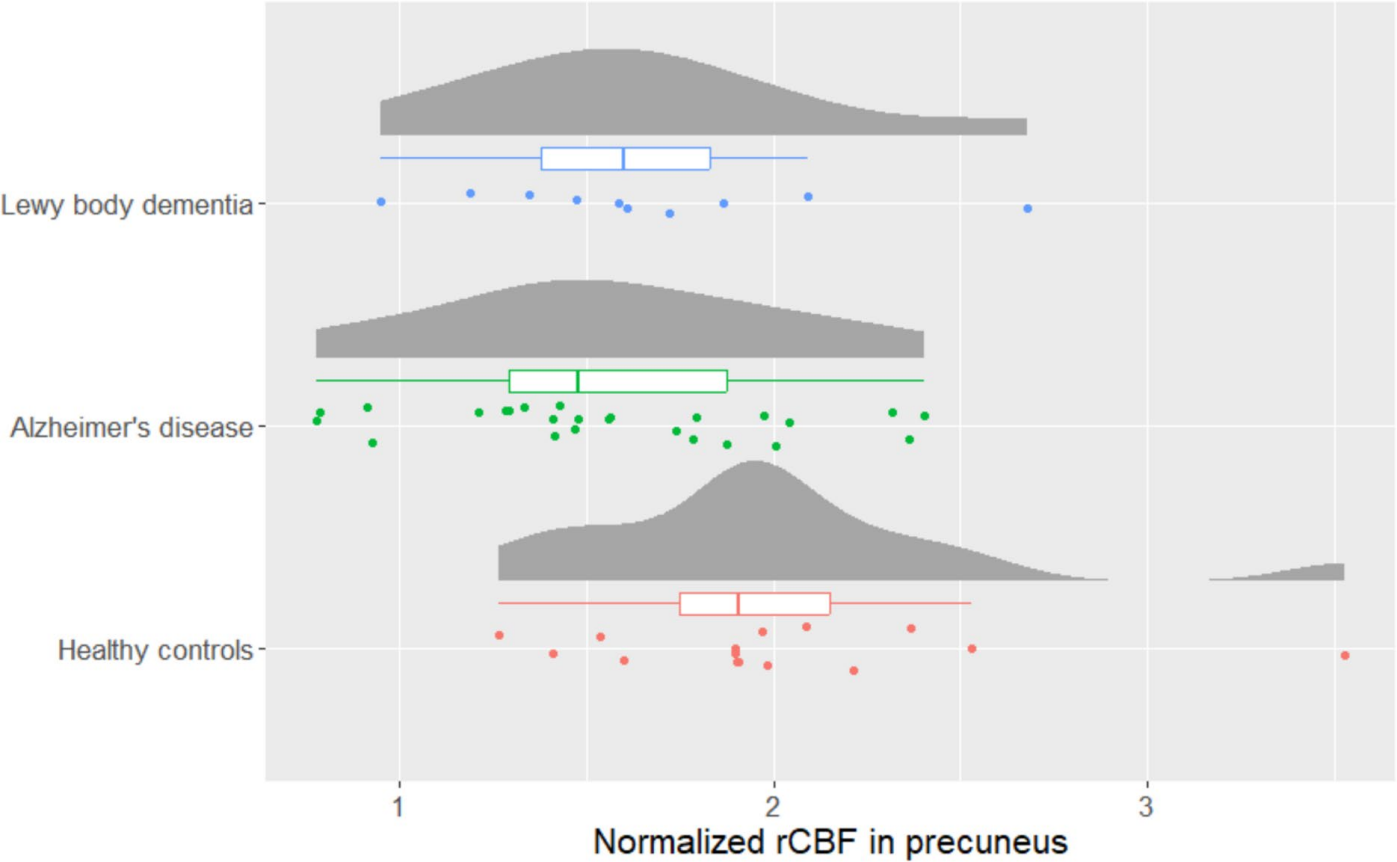
Raincloud plot showing the normalized regional cerebral blood flow (rCBF) in the precuneus for patients with Lewy body dementia (*n* = 10), Alzheimer’s disease (*n* = 25), and healthy controls (*n* = 15). The boxplots indicate the median, interquartile range while the whiskers extend to the largest and smallest data points within 1.5 times the interquartile range from the third quartile and first quartile, respectively. A significantly lower rCBF in precuneus was found between Alzheimer’s disease and healthy controls (*p* = 0.023)

A significant difference in the normalized (to the global CBF) rCBF in precuneus was found between the groups (*p* value: 0.028, *F*-value: 3.85). The post hoc analysis found a significant difference between AD and HC (*p* value: 0.023) (Fig. 5). However, we did not find a significant association between lactate and normalized rCBF in the precuneus (estimate: 0.03, *p* value: 0.44).

## Discussion

We anticipated finding signs of tissue hypoxia manifested as elevated cerebral lactate concentration and reduced CMRO_2_ across both AD and DLB patient groups. We did find reduced rCBF in the precuneus of AD patients and reduced global CBF in DLB patients suggesting a possibility of hypoperfusion-induced hypoxia [50, 51]. However, this was not accompanied by reduced CMRO_2_ or elevated lactate concentration. Since lactate levels were similar across groups and unrelated to rCBF, the reduced perfusion is more likely due to neuronal loss. Neuronal loss can also be indicated by lower NAA concentrations, with our study showing significantly lower NAA levels in the precuneus of AD patients compared to DLB, supporting previous findings of reduced NAA in AD [52, 53]. The reduced CBF in the patients is likely a result of vascular burden, as suggested by the association between lower global CBF and higher Fazekas scores. This could explain the reduced global CBF observed in DLB. Although DLB may not directly be associated with increased vascular burden, a recent multicenter study found that DLB patients had higher Fazekas scores than those with Parkinson’s disease or mild cognitive impairment [54]. Also, a previous study has found that patients with DLB and autonomic dysfunction show increased small vessel disease pathology, which contribute to WMHs and reduced CBF [55]. Furthermore, dopaminergic degeneration, a distinct feature to DLB, has previously been associated to periventricular WMH in patients with DLB [56]. The previous findings, together with our results, support the notion of cerebral vascular pathology being present in DLB.

In contrast to the finding in our study, two prior studies found higher brain lactate in the brains of AD patients using MR spectroscopy [18, 19]. The cause for these discrepancies is not obvious. The studies included comparable numbers of patients as our study, suggesting that differences in statistical power due to sample size may not fully explain the findings, though the small sample sizes across studies remain a limitation. The discrepancy might stem from differences in MRS methodologies, where the earlier studies employed sequences not specialized for lactate, but rather for a broader range of metabolites. By using these sequences, other metabolites and especially lipid molecules in the brain will affect the lactate estimate. Our study employed an MRS sequence optimized for lactate detection using a long echo time, thus reducing interference from other compounds.

The results of this study challenge the hypothesis that tissue hypoxia is a driver of pathology in neurodegenerative diseases. Our findings indicate that in the patients with AD, both oxygen metabolism and global CBF are preserved when accounting for underlying brain atrophy. When these physiological systems remain intact, they may not represent effective therapeutic targets. In contrast, the patients with DLB showed hypoperfusion, which may represent a potentially distinct vascular contribution to the pathology in this group. These observations highlight the need for a deeper understanding of the heterogeneity of dementia pathology observed between patients, for example identifying which patients exhibit hypoperfusion and which do not.

The failure to detect differences in CMRO_2_ or lactate concentration between patients and controls in the present study could also be related to the disease stage, as it has previously been shown that the metabolic deficits in dementia compared to controls vary throughout disease progression. One study using FDG-PET and structural MRI in AD patients found more pronounced changes in FDG-PET than MRI in early stages, with the reverse trend in later stages [57]. A large meta-analysis, however, indicated that certain regions are structurally affected while others experience more metabolic changes [58]. Another voxel-wise study reported that hypometabolism often exceeds atrophy in key areas, notably the precuneus [59]. Thus, it is possible that metabolic changes are more marked in early disease stages, potentially explaining the lack of differences in oxygen consumption and lactate in our study. Another important possible explanation could be that cortical hypometabolism primarily reflects structural changes [60]. Hypometabolism findings may therefore indicate reduced brain volume due to partial volume effects [61]. Although previous studies showed that the precuneus is affected in both AD and DLB, the variability in hypometabolism patterns measuring glucose consumption on FDG-PET [62] may reduce the sensitivity to detect metabolic changes. Future studies should combine FDG-PET with MR spectroscopy to clarify this association.

The study has a number of limitations. Most importantly, we did not perform FDG-PET scans at the time of the MRI scan, which would have provided information on the location of potential hypometabolism. This would ideally be performed with quantitative FDG-PET of the brain to be able to directly compare the two modalities [63]. In the current study, the sample size was low and especially for patients with DLB, which due to heterogeneity in disease progression and hypometabolism patterns may have affected the findings. A larger group of patients is needed to fully understand the association changes in brain metabolism.

## Conclusions

We did not observe evidence for affected cerebral oxygen metabolism in either AD or DLB patients assessed by measurement of CMRO_2_ and cerebral lactate concentration. The observed hypoperfusion in patients is likely due to neuronal damage from the underlying pathological process and does not cause hypoperfusion‐induced tissue hypoxia. Concurrent MRI and FDG-PET in different stages of the disease is needed to understand the association between hypometabolism and cerebral oxygen consumption and lactate production.

## Disclaimer

None of the funding parties had a role in the collection, analysis, and interpretation of data or in the writing of the manuscript or whether to publish the results of the study.

## Data Availability

Software used for calculating CBF by the PCM technique is available at https://github.com/MarkVestergaard/PCMCalculator/. Software used to calculate SvO2 from the SBO technique is available at https://github.com/MarkVestergaard/SBOCalculator/.
